# Using a virtue ethics lens to develop a socially accountable community placement programme for medical students

**DOI:** 10.1186/s12909-019-1679-7

**Published:** 2019-07-05

**Authors:** Mpho S. Mogodi, Masego B. Kebaetse, Mmoloki C. Molwantwa, Detlef R. Prozesky, Dominic Griffiths

**Affiliations:** 1 Department of Medical Education, Faculty of Medicine, University of Botswana, Gaborone, Botswana; 2 Division of Studies in Education, Wits School of Education, University of the Witwatersrand, Johannesburg, South Africa

**Keywords:** Social accountability, Community-based education, Community-engaged medical education, Community-oriented medical education, Virtue ethics, And community placement

## Abstract

**Background:**

Community-based education (CBE) involves educating the head (cognitive), heart (affective), and the hand (practical) by utilizing tools that enable us to broaden and interrogate our value systems. This article reports on the use of virtue ethics (VE) theory for understanding the principles that create, maintain and sustain a socially accountable community placement programme for undergraduate medical students. Our research questions driving this secondary analysis were; what are the goods which are internal to the successful practice of CBE in medicine, and what are the virtues that are likely to promote and sustain them?

**Methods:**

We conducted a secondary theoretically informed thematic analysis of the primary data based on MacIntyre’s virtue ethics theory as the conceptual framework.

**Results:**

Virtue ethics is an ethical approach that emphasizes the role of character and virtue in shaping moral behavior; when individuals engage in practices (such as CBE), goods internal to those practices (such as a collaborative attitude) strengthen the practices themselves, but also augment those individuals’ virtues, and that of their community (such as empathy). We identified several goods that are internal to the practice of CBE and accompanying virtues as important for the development, implementation and sustainability of a socially accountable community placement programme. A service-oriented mind-set, a deep understanding of community needs, a transformed mind, and a collaborative approach emerged as goods internal to the practice of a socially accountable CBE. The virtues needed to sustain the identified internal goods included empathy and compassion, connectedness, accountability, engagement [sustained relationship], cooperation, perseverance, and willingness to be an agent of change.

**Conclusion:**

This study found that MacIntyre’s virtue ethics theory provided a useful theoretical lens for understanding the principles that create, maintain and sustain CBE practice.

**Electronic supplementary material:**

The online version of this article (10.1186/s12909-019-1679-7) contains supplementary material, which is available to authorized users.

## Background

Current trends in community-based education (CBE) challenge educators to pay as much attention to students’ learning of ‘heart’ knowledge as they do to their ‘head’ knowledge [1–3]. One such approach is community-based education (CBE), which involves educating the head (cognitive), the heart (affective) and the hand (practical) by utilizing tools that enable us to broaden and interrogate our value systems [3]. We refer to ‘community placement (CP)’ programmes as well as ‘community-based education (CBE)’ programmes where CP programmes define particular examples CBE for health professions students. In their quest to develop and implement effective CBE programmes, most medical schools are increasingly turning to socially accountable learning programmes [4–7]. In fact, the World Health Organization recommends social accountability as a requirement of medical schools to guide their teaching, research and service activities towards meeting the most important health needs of the communities that they serve [5, 8]. Increasingly, medical training institutions globally are using the social accountability framework to design their curricula [5, 9–15] as a way to prepare future doctors better to be socially accountable and responsive to the needs of the community [16].

An important aspect of social accountability calls for medical schools’ CBE practitioners and communities to jointly identify community needs [13]. The practice of involving the community in medical education has evolved from community-oriented medical education (COME) [17] to community-based medical education [18], and most recently to community-engaged medical education (CEME) [19]. In COME, the subject matter studied by the students has direct significance to the priority needs of the community although with limited learning activities in the community [17]. In CBE, besides considering priority needs of communities, there are consistently scheduled learning activities taking place in the community. However, the medical school passively involves the community [20]. In CEME, not only are the priority needs considered and activities consistently scheduled but also the community is actively involved [19].

As mentioned, though many medical schools are increasingly adopting socially accountable learning programmes it is often not clear what ethical principles should underpin them, and whether these are adequate. There is increasing apprehension that future doctors are not well prepared to respond sufficiently to wider determinants of health, health inequalities, and the needs of susceptible individuals and societies at large [21, 22]. Furthermore, there is also the growing recognition that the holistic practice of medicine must go beyond modern dominant utilitarian and principle-based approaches [23–25]. MacIntyre’s virtue ethics (VE) theory offers an approach to conceptualising a socially accountable CBE programme. It also offers a valuable way of theorizing how a CBE programme could resist and reverse the fragmentation of modern medicine, by establishing the sense of moral community that constitutes the practice of medicine, and highlighting the values and virtues that are central to the holistic nature of this practice. Furthermore, the theory allows trainee doctors deeper insight into how their goals and the internal goods that are central to the practice of medicine could sustain and enrich their own lives and the lives of the community they serve.

The purpose of this article then is to report the findings of a secondary and deeper analysis of the data from a qualitative study. This study was initially conducted to gain insight from key informants regarding the type of socially accountable community placement programme needed for first- and second-year undergraduate medical students, and how to develop and implement it. After the initial research, we found that the rich data abounded in ethical expressions. We therefore decided to conduct a secondary analysis informed by MacIntyre’s virtue ethics theory. The research questions driving this secondary analysis are the following: what are the goods which are internal to the successful practice of CBE in medicine, and what are the virtues that are likely to promote and sustain these goods?

### Ethical considerations

Ethical approval of the study was obtained from the University of Botswana Institutional Review Board, Ministry of Health ethics committee (Permit: E1/20/2 XXII (7) and Ministry of Education ethics committee (PPME 13/18/1 PS V (228). Each participant consented to participate in the study after the study procedures, risks and benefits were shared with him or her.

## Methods

In reporting the study findings, we used the Consolidated criteria for reporting qualitative research (COREQ) checklist [26] as a guide. We conducted a secondary theoretically informed thematic analysis of the primary data obtained by conducting key informant interviews. The thematic analysis was based on MacIntyre’s virtue ethics theory. We identified categories, themes and codes from the data [27].

### Setting

The Bachelor of Medicine and Bachelor of Surgery (MBBS) programme at the University of Botswana enrolled its first cohort of students in 2009. The medical school was started to address the shortage of citizen doctors in the country. Botswana has faced a serious shortage of doctors for many years. This shortage has been worsened by extremely high rate of infectious diseases, the ongoing burden of developing chronic, non-communicable diseases, and the emigration of medical doctors. An attempt was made in the 1990s to train young Batswana^1^ at medical schools around the world, however, most of those medical graduates did not return home. It was because of this that the country decided to inaugurate a medical school [28]. Furthermore, it was hoped that local training of doctors would be done close to communities so that their needs could be considered when health services are provided.

The medical school selected the model of a 5 year outcomes-based, problem-based, socially accountable, community-oriented, theme-based, and spiraling programme [28]. In the first 2 years (Phase I), students concentrate on the biomedical sciences and basic clinical and communication skills, and they experience patient contact through weekly clinical placements. The last 3 years (Phase II) are the clinical years where students complete five eight-week rotations each year [28]. CBE is integrated in both Phases I and II of the programme. In the Phase I of the programme, CBE is aimed at sensitizing students to the health determinants of the community they serve [28]. Thus, the curriculum is intended to be socially accountable by addressing the country’s health care needs.

### Study team and reflexivity

The study team was composed of five academic staff: four (MSM, MBK, DRP and MCM) from the Department of Medical Education (DME) at the UB FoM and one (DG) from the University of Witwatersrand. DRP, MSM, and MCM are medical doctors and health professions educators. MBK is an instructional designer and educational developer. DG is a philosophy lecturer and conceived the conceptualization and application of a virtue ethics lens to the study. At the time of the study, MSM was teaching public health and was responsible for implementing the community placement programme. She is also the principal investigator and conducted all interviews. At the time of data collection, some of the participants were colleagues of the principal investigator.

### Study population

Sampling was purposive, attempting to include a range of key informants in CBE in the local setting. Numerous key informant participants from diverse backgrounds were initially sampled. They comprised of the dean and associate dean of the then faculty of health sciences, heads of the schools of medicine, nursing, public health, associate programme directors of family medicine and internal medicine and, heads of community nursing and environmental health. Deputy principals of the eight Botswana health training institutions and their faculty members were also included. Also invited were the health sciences librarian, Information technology officer, a health service provider who could either be retired or currently serving, health professions graduates within 1 year of graduation, curriculum experts, and educationalists at the university or local school that have learning programmes in the community or any other person involved in community work.

In the end only eight of the targeted key informants were interviewed, mostly for logistical reasons (e.g. delays in obtaining ethical clearance from other institutions, availability for a long interview, and the school’s high attrition rate [28]). Those interviewed included three health professions educationalists, two general educationalists, one instructional designer, one community worker, and one former Ministry of Health official. One participant referred another participant (snowballing effect), believing that this new participant could provide useful information about what we were interested in learning.

### Interview setting

Of the eight participants who were interviewed, seven were interviewed at their offices and one preferred to be interviewed at the interviewer’s office. There were no other people present during interviews besides the interviewer and the participant.

### Data collection

Data were collected by means of in-depth semi-structured interviews. The study was explained and participants signed a consent form (Additional file 1: Appendix A). The interview guide (Additional file 2: Appendix B, summarised version in Table 1) probed their knowledge, experience and expectations of a CP programme as well as their recommendations on what would inform the development of such a programme. The interviews were conducted in English and lasted from 20 to 45 min each. An audio recorder was used with consent and audio-recorded data were subsequently transcribed verbatim. Field notes were also made during the interviews. Transcripts were not returned to participants for comment or correction; although attempts were made, the logistics proved too challenging.

**Table 1:** Socially accountable CBE interview guide: summary of relevant questions posed

1. What do you consider as social accountability in your programme?	
2. How do you design the learning outcomes to be achieved, what should they focus on?	
3. What kind of educational activities will ensure students become socially accountable?	
4. What kinds of resources are needed for this kind of programme?	

### Data analysis

The primary analysis did not produce new insights into the practice of CBE. The researchers felt strongly that the data contained richer insights, leading to further reading and discussion. We came across the theory of virtue ethics and proceeded with a secondary thematic analysis of the data using MacIntyre’s theory of virtue ethics’ concepts as guiding themes: the internal goods inherent in the practice of CBE, and the virtues needed to sustain them [24]. In selecting virtue ethics as a framework we accepted that there may be other frameworks (e.g. ‘human rights’) which also provide valuable insights into the practice of CBE. MBK, MCM, MSM and DRP analysed the data. We selected and reviewed one transcript to familiarize ourselves with the data [27], then each coded the same transcript independently and discussed the codes to ensure that our understanding and coding were consistent [27]. We all then read the other seven transcripts and coded them [27], following which we met to discuss each transcript to come to a consensus about emerging codes. We collated the codes from all eight transcripts around broad themes (related to MacIntyre’s theory) that came up during our discussion sessions. MSM, MBK and MCM then interrogated the broad themes of ‘internal goods’ and ‘virtues’ further to generate sub-themes [27]. We checked the codes twice to make sure that they were relevant to the themes and that codes within each sub-theme were related to the same concept.

## Results

Themes related to design and implementation of a socially accountable community placement programme emerged. We report on two broad themes: goods internal to the practice of CBE, and virtues needed to enable and sustain them.

### Internal goods for effective community placement

This theme describes internal goods to be realized and achieved for an effective CP programme. For excellence in the practice of community placement, faculty, students and the community should be aware of such goods and the importance of achieving them. The following internal goods emerged from the study: a service-oriented mind-set, a deeper understanding of community needs, a transformed mind, and a collaborative approach.

#### A service-oriented mind-set

Participants considered it important for students to have a heart for service. That is, students to be re-oriented from self-centeredness, and might be challenged to look outside of themselves.

*Too often adolescents were told, go play your sports, go do your studies. It’s all about self, self and the point of service is have them come out of themselves* (8:46–48).

In fact, faculty should purposefully challenge students towards service orientation:

*I am now teaching them about what service means, why we should be service-oriented why we should do service* (2:89–91).

There are many opportunities for service, including schools, home-based care, and clinics through which students can be engaged.

*Talking about your students, … during their placement … they can just go to schools and even talk about hygiene and this would be a very useful exercise to remind them that even as clinicians in future they still continue what they already started* (5:135–140).

*These students need to be exposed to the concept of home-based care* (7:28).

*They go to the health clinics … where we expose them to primary health care concepts. Most importantly, they learn the socio-cultural aspect as they interact with the community and other stakeholders* (7:14–17).

Additionally, service should not just be viewed as benefiting the community only; students also can benefit personally from the service with which they engage. Service can be utilized as a way of developing student leadership:

*It’s now quite fashionable to have service as part of your curriculum. Many top universities are not exclusively focusing on exam results. They want some evidence the students they are considering admitting have a commitment beyond themselves. Other top universities in the world say show us what you care about and what have you done, what leadership have you exercised in service activities, how have you changed other people’s lives? How durable has your relationship been?* (8:113–118).

#### A deeper understanding of community needs

In order to serve the community effectively, one has to understand and be in tune with the needs of the community being served. Students and faculty have to engage with the community and be exposed to people who live lives that are less privileged than theirs:

*[A] ll we can do is expose them to situations where it is possible for them to form relationships* (8:30–31).

*Students go around an area and get exposed to a variety of cases. You get destitutes, you get vulnerable children, you get orphans, and you get emergency cases. So there is a whole range of different experiences* (2:324–326).

*You can walk over the road just over there and you will find poverty on a massive scale, you just need to know where till you find it* (2:288–291).

*Students have a lot to learn because most of them come from intact families, most of them come from positions of wealth and privilege* (8:11–13).

A community needs assessment is viewed as the exercise that would enable students and faculty to assess and understand the actual needs of the selected community. This would also ensure that the CP programme is aligned to the actual needs identified in the community:

*[J] ust have to do a needs assessment of the community so that the curricular is also aligned to the needs of the community* (3:10–12).

*[A] nd I would be saying go out there find a need in the community and work with those, partner with them* (3:195–196).

*[W] hat the needs of the population are … so what are the unknowns – about the health in the community … why are our children dying of malnutrition for example* (1:29, 32–34).

Beyond understanding the needs of the community, there is need to find ways to address such needs and empower the community.

*I’ve managed to get access to Riverwalk [mall] to sell their wares, they did a show; some things sold out some things didn’t but they felt such a sense of empowerment to have access to a market* (2:205–207).

*So what we did was work to get her a plot of land and build her a small structure on it that she can rent out. From the rental income she would be able to … buy the formula that the child needs, and to have somebody watching her kid while she goes off to work. It’s very practical, very problem solving-oriented at its best* (8:202–206).

#### A transformed mind

Community placement should engender transformation and attitude change towards service orientation.

*I tell stories to privileged kids about what life is like for those who are not privileged and ask them to change their attitudes, change their views and change this country* (2:48–50).

*Having completed this course, in what ways would you want the student thinking differently, in what ways would you want them acting differently, and in what ways you would want them feeling differently?* (3:152–154).

Such transformation should lead to a connectedness with those being served:

*The overriding objective is to connect with and form a bond with this person in the community (*8:169–171).

*The emotional connection is far more than the academic rationale. You know these people need help, it’s putting a name to a face and forging [an] emotional connection that tends to sink in deeply* (8:40–43).

*Suddenly, they find themselves connecting on deep and regular level with people that they don’t have their same advantages* (8:50–53).

Furthermore, such transformation should cultivate professional behaviour, which students should exhibit during community placement:

*We are to produce leaders who are never afraid to mix the cow dung and the dirt, who never lose edge to rub shoulders with the poorest in the land* (8:107–109).

*[N] ot being afraid to make mistakes, but when they are made [mistakes] being willing to learn from them, and to keep picking yourself up and dusting yourself off and giving it another try from another different angle* (8:235–238).

*We really want our doctors to be good communicators to realize that health is a partnership between the patient and the health care provider* (6:24–25).

#### A collaborative approach

Additionally, collaboration and inter-professional partnerships can enable different groups to work together in providing service to the community. For instance, there is need for CP programmes to collaborate closely with community groups:

*[I] n some other countries they basically work with community leaders, student reps, village committees* (4:96–97).

*[L]ong-term partnerships with a variety of community activists and people who are working to address problems of poverty* (8:7–9).

There is also need for students to engage with other health professions students to serve the community better:

*[I]t also happens at student level: medical students, lab students, public health students, any health professions students they are taught together* (4:128–130).

Moreover, participants suggested a collaborative platform for experts and practitioners of CBE programmes:

*I’d like to have a workshop or conference calling in everyone known to be doing any kind of work in this … kind of addressing the needs of the communities; call all together and I’m talking social services, police, doctors, health care workers* (2:265–258).

*[H]ow we can be coordinated and work together as a cohesive team of experts* (4:125–126).

### Virtues needed for effective community placement

To realize the above-mentioned goods internal to an effective socially accountable CBE programme, faculty and students should possess some virtues that enable then to sustain the practice of CBE. The following virtues emerged from the study: empathy and compassion, connectedness, accountability, engagement (sustained relationships), cooperation, perseverance and willingness to be an agent of change.

#### Empathy and compassion

Those involved with community-based education should be compassionate, credible, and empathetic for it to be effective.

*In terms of Batswana future leaders which we are hoping to help create, it’s important for future leaders to be well aware from personal experience. For instance, the orphan crisis in Botswana, that segment of the population between a quarter and a third of Batswana living in poverty* (8:13–17).

*If I haven’t touched those people myself, I have no credibility … I go visit someone dying in Old Naledi (2:258, 260)*

#### Connectedness

Besides being compassionate and empathetic, one of the participants strongly suggested that those involved with community-based education should have a genuine emotional connection with the community.

*The emotional aspect of it matters. You know, building that human relationship* (8:171–172)

*It’s an emotional connection far more than the academic rationale. You know these people need help; it’s putting a name in a face and forging emotional connection that tends to sink in deeply* (8:40–43).

*You’re going to give them a chance to feel the emotional connection that you’ve got. So you got to show pictures of the situation, you going to talk about this person, you are going to make this come alive in the hearts of your fellow students* (8:261–264).

*Suddenly they find themselves connecting on deep and regular level with people that don’t have their same advantages* (8:50–53).

#### Accountability

It is also important that those engaged in CBE have a sense of responsibility and accountability to the community:

*They do have a responsibility to the whole of society … and the privileges they are given by expensive medical course, their parents educating them, the university educating them, and the respect that doctors are held in. It all means that they have an on-going responsibility to help the people in society, all people in the society, not just those who can pay the fees* (1:41–45).

*The school must have a social accountability dimension, must be accountable to the people it tries to serve* (1:6–7).

*We have the same standards of accountability of everything you would expect of a business in this work that we do* (2:17–18).

#### Engagement

Beyond a sense of responsibility, CBE participants should actively engage with the community, for instance, by maintaining long-term activities and services:

*To this day, we have maintained that relationship with Gabane and to that we have added a number of other services* (8:100–102).

*Keep staff engaged and supported* (8:129).

*If you have long-term teaching staff they can help connect the students and sustain the relationships* (8:132–134).

*We have added at least three different programmes in Old Naledi. We work with Camp Hill School out at Otse, we work with churches or homes, and we go to [the] Paediatric ward at Princes Marina. We are engaged in SOS Children’s Village* (8: 102–105).

*We need to respond first because someone’s house is burnt down, something is flooded, a baby is been dumped, they’ve got no nappies [diapers], no formula, they’re struggling to put something together; am giving you the realities for resource stretched around, social workers on the frying pan* (2:185–188)**.**

Additionally, there is need to engage enthusiastically and be open to new possibilities:

*Teenagers especially have a great energy and solutions* (2:56–47).

*Projects fall on my lap, things turn out. People phone me to say that we’ve heard about this or that. I get things somehow, seems like a magnet … So we’re always open to adventure* (8:188–191).

Additionally, participants considered it important for those participating in CBE to engage the community with humility.

*And there are other people who are doing things; I mean it’s not just me* (2:476).

*If we are to produce leaders, they must be leaders who are never afraid to mix the cow dung and the dirt, who never lose edge to rub shoulders with the poorest in the land* (8:107–109)**.**

*We don’t care who gets the credit because that’s how we get work done* (2:28).

#### Team player

Participants mentioned that working together at individual and institutional levels is vital for effective community-based education.

*They are interested in bringing together all organizations and actually saying let’s really understand what the problems are before we jump into conclusions* (2:274–275).

*When I am saying health professionals I am not referring to doctors, I am saying the entire team. The nurses that will be there should be able to advise the students, the pharmacists that will be there should do the same thing* (4:51–53).

#### Perseverance

One of the participants emphasized that effective engagement with community-based education calls for perseverance, where one does not give up during the course of the project:

*It’s very difficult, very difficult, but it’s just rebooting every week. After that it’s the same message again, and again, and again* (2:431–432).

*There’s nothing worse than making a promise, doing something, then stopping it because you are either bored with it or it’s too much trouble or it’s a headache … that is the most common thing … That people kick start [something], they are all enthusiastic, then after a few challenges in the future they stop or something changes in their life, they stop it. You know, this is not something you can switch on and off* (2:458–463).

*If you implement something, implement it. Get the thing in your systems and make it happen. That is what I really try and focus on* (2:421–423).

#### Willingness to be an agent of change

Ultimately, the person engaged with CBE should be willing to be an agent of change. That is, he or she should be willing to do something about the challenges faced by others. Participants seem to suggest that being an agent of change involves being introspective and reflective:

*I am saying we need to probably step back a little and ask ourselves, where do we want to go as a nation?* (5:105–106).

*We’ve got to be doing something, we’ve got to be feeling something, and yes, you should reflect on it and figure out your next step* (8:72–73).

Additionally, such an agent of change needs to possess transformational leadership qualities:

*We have this opportunity to lead the faculty of health sciences, but we can only do that if really we open up ourselves to feel that we are part and parcel of the fact and this is going to alleviate a lot of issues* (4:219–221).

*You know, in hospitals you have a ready audience; you have children who are young who still don’t have cancer of the cervix. Would this not be a better time that as you give them treatment for pneumonia, you start talking to the parent and you say to them, you know there is something known as cancer of the cervix and you need to make sure that when you child is nine years old she is vaccinated against the human papilloma virus [HPV]. And when you do that thing you are beginning to change the thinking pattern of the parents* (3:124–132).

*Transform their idea of who they can be and how they can serve their communities* (8:54–55)**.**

Furthermore, there is a need to think innovatively:

*There need to be different ways of thinking about things* (2:360–261).

## Discussion

In order to situate and fully reflect on our findings it is necessary to give a brief overview of MacIntyre’s virtue ethics theory [26–28]. For MacIntyre, virtues are dispositions which not only sustain practices, but also help us achieve goods internal to these practices [29]. It is through our engagement with and commitment to practices, in this case CBE, that we can realize an ethical, good life. Internal goods are partially definitive of each particular practice and represent the standards of excellence associated with it. These goods, MacIntyre writes, are “the rewards of the virtues” whose achievement “is a good for the whole community who participate in the practice” ([30] p190,198) As our study’s results demonstrate part of the purpose is to identify what some of these internal goods could be for effective student community placement in CBE.

As MacIntyre suggests vital to the flourishing of practices such as CBE are the communities in which they are exercised. One such communal space is medicine, which Pellegrino and Thomasma argue should ideally constitute a “moral community” [25]. They argue that physicians are bound together by a common moral purpose, namely a commitment to care for the sick, and this purpose necessitates a set of shared and collective moral obligations consistent “with the ends, goals, and purposes of medicine” ([25] p3,32,45). However, a variety of factors, such as the increasing specialization of modern medicine, the profit motive and regulatory pressure, have caused modern medical practice to become fragmentary and dispersed, often distant or isolated from the communities they are meant to serve and heal. This fragmentation promotes values such as individualism and self-interest, which are inimical to the kinds of moral obligations physicians share as a community [25].

Thus two significant motivations for CBE for trainee doctors are, firstly, to establish this sense of belonging to a moral community and, secondly, to promote a deep recognition of the kinds of virtues which characterize the practice of medicine in the broader communal space. While various benefits of CBE are well-documented, these tend to be broadly utilitarian in focus, attentive to tangible outcomes, for example improved service delivery or increased student familiarity with the health care system [31]. In contrast, this study uses virtue ethics is used as a lens for reflecting on how to develop a socially accountable CP programme for first- and second-year undergraduate medical students.

We identified goods internal to the practice of an effective CP programme and the virtues that participants of such programmes should possess and exhibit to drive a socially accountable programme.

As discussed in the ‘Background’ above, most CBE programmes include considering community needs [4, 7, 32]. However, these community needs should not be taken into account by the medical school on its own, without active participation of the community [13, 33, 34]. Instead, medical schools should treat the community as an equal partner in most aspects of the CBE programme [9, 35, 36]. Our data suggests that a thorough understanding of societal needs is an important good internal to an effective socially accountable CBE. We suggest that such thorough understanding of community needs is pivoted on the training institution and the community having a balanced partnership [31]. Participants engaged with CBE should not only be tangentially exposed to those in the community but they should also be intimately involved with the needs of those who are less privileged than themselves [36]. To have and sustain a deeper understanding of the community, CBE participants need to have the virtues of emotional connectedness and engagement.

It is not enough to understand community needs; trainee doctors also need to be convinced and willing to do something about such needs. CBE programmes have been shown to instil in students the desire to implement sustainable service activities [37, 38]. Our data indicates that a service-oriented mind-set is a critical element of an effective CBE programme. CBE programmes should intentionally challenge students towards service orientation and provide opportunities for such service. Empathy and compassion are required to drive participants to respond to community needs that have been jointly identified. Additionally, participants should engage with the community through sustained relationships.

In as much as the trainee doctor is providing service, it is necessary to recognize the importance of collaborating with other professionals and institutions for seamless and effective service provision. There is evidence from the literature that CBE is conducted across different health profession disciplines [36]. As such, a collaborative multidisciplinary approach to CBE could potentially be more effective than non-cooperative single disciplines, which our data suggests. This allows for a coordinated and cohesive effort in providing service to the community. Furthermore, such collaboration provides an opportunity for students to gain early exposure to the students and professionals of other health professions. A collaborative approach to CBE requires each trainee doctor to be an engaging team player who is willing and able to reach across to others at individual and institutional levels.

Ultimately, a transformed mind-set is the goal that should be achieved through the CBE experience. Transformation can either be evolutionary over time or revolutionary following a disorienting experience [39–43]. Our data suggests that transformed participants should experience a change in attitude and in the way they think about community needs and engagement, and collaborate with others towards service delivery. The willingness to be an agent of change seems to be the overarching human quality needed to achieve excellence in the practice of a socially accountable CBE. Agents of change should possess other virtues, such as perseverance and accountability, in their quest to serve. That is, they should have a sense of responsibility to the community and be willing to keep going despite challenges they might face.

### Limitations of the study

Our decision to interview curriculum experts, educators and community workers limited our study in that it lacked the voices of students and the community. This tended to provide more guidance from experts in the design and development of a CBE programme with limited community guidance on their priority needs. The lack of a community voice could potentially result in an incomplete picture of a truly balanced CBE partnership: our use of institutional experts could potentially diminish cultural aspects of CBE. Similarly the lack of a student voice could lead to CBE programme design that fails to take into account the goods and virtues that students might ascribe to CBE, which may well be different from those of the ‘experts’. Subsequent research could complete the picture by investigating the applicability of these new perspective by interviewing community members and students, and thus get to hear the community and student voices regarding an effective CBE programme.

Regarding reflexivity, the main limitation appears to be that the eight came from a relatively limited professional background of educators who were likely sympathetic to their colleague wanting to do research. Thus the uniformity of themes may reflect this. More diverse themes that do not fit so well with VE may have resulted from a more diverse sample. If the primary investigator is passionate about community based education issues, this is likely to lead to biased way of interviewing and thus biased responses. This could be addressed by ensuring that the interviews were as diverse as possible.

## Conclusion

This study found that MacIntyre's Virtue Ethics theory provided a useful theoretical lens for understanding the principles that create, maintain and sustain CBE practice.

## Additional files

Additional file 1: Consent form. (DOCX 18 kb)

Additional file 2: Interview guide. (DOC 120 kb)

## Data Availability

The data supporting these results can be requested from Dr. Mpho Mogodi at the following email addresses: mogodim@ub.ac.bw; mpho910@gmail.com

## Abbreviations

CBE: Community Based Education
CEME: Community Engaged Medical Education
COME: Community Oriented Medical Education
CP: Community Placement
CPP: Community Placement Programme
MBBS: Bachelor of Medicine and Bachelor of Surgery
SA: Social Accountability

## Footnotes

1 Citizens of Botswana
