# Supplementary material for: Using a virtue ethics lens to develop a socially accountable community placement programme for medical students
Source: BMC Med Educ. 2019 Jul 5;19:246. doi: 10.1186/s12909-019-1679-7 (PMC6612096; doi:10.1186/s12909-019-1679-7)
Supplement: Supplementary file 1 — Consent form. (DOCX 18 kb) [file 12909_2019_1679_MOESM1_ESM.docx]

Dear Study DEa Dear Study Participant,

You are being invited to take part in a research project. Please take some time to read the information presented here, which will explain the details of this project. Please ask the researcher any questions about any part of this project that you do not fully understand. It is very important that you are fully satisfied that you clearly understand what this research entails and how you could be involved. Also, your participation is entirely voluntary and you are free to decline to participate. If you say no, this will not affect you negatively in any way whatsoever. You are also free to withdraw from the study at any point, even if you do agree to take part.

One of the many areas in health professions education is ***socially accountable*** medical/ health professions education. This is our way of producing health professionals who are aware of, concerned about and are willing to address the needs of communities they serve. This research study has been approved by the ethics committee. We need to obtain consent from all participants who will participate.

The World Health Organisation (WHO), defines Social Accountability of medical schools as “the obligation to direct their education research and service activities towards addressing the priority health concerns of the community, the region, and / or the nation they have a mandate to serve. The priority health concerns are to be identified jointly by governments, health care organisations, health professionals and the public.”^[[1]](#footnote-1)^

Part of what you do as a health professionals’ educator is to develop field attachment programmes for your students to go and interact with their communities. We would like to request you to share your insight regarding such an experience. We are interested in steps you follow to ensure you develop relevant learning objectives for your students. What methods do you employ in assuring your students get good grasp of the fundamental concepts involved in engaging communities in health care.

The survey is completely voluntary, and if you do not feel inclined to complete the survey.

Thank you very much for taking the time out of your busy schedules to participate in this survey.

*

I declare that:

• I have read or had read to me this information and the consent form and it is written in a language that I understand and am comfortable with.
• I have had a chance to ask questions and all my questions have been adequately answered.
• I understand that taking part in this study is voluntary and I have not been pressurised to take part.
• I may choose to leave the study at any time and will not be penalised or prejudiced in any way.
• I may be asked to leave the study before it has finished, if the study doctor or researcher feels it is in my best interests, or if I do not follow the study plan, as agreed to.

. I have been given a copy of the consent form to keep.

I …………………………………………………………….. (Name of Participant), Faculty member

(UB-FHS) / Health Professional / ____________________________________(specify any other role in the Botswana Health System) fully consent to participate on this study on **Developing a rural placement programme that is *socially accountable* for first and second year UB SOM medical students.**

In case you may have questions and concerns that arise later after the administration of the survey, kindly contact the Primary Investigator of the Survey:

Dr. Mpho S. Mogodi

E-mail: [mpho.mogodi@mopipi.ub.bw](mailto:mpho.mogodi@mopipi.ub.bw); [mpho910@gmail.com](mailto:mpho910@gmail.com)

Telephone: +267 355-4552

1. Boelen C, Heck JE. Defining and measuring the social accountability of medical schools. Geneva: WHO; 1995 WHO document WHO/HRH/95.7 [↑](#footnote-ref-1)
