# Supplementary material for: Using a virtue ethics lens to develop a socially accountable community placement programme for medical students
Source: BMC Med Educ. 2019 Jul 5;19:246. doi: 10.1186/s12909-019-1679-7 (PMC6612096; doi:10.1186/s12909-019-1679-7)
Supplement: Supplementary file 2 — Interview guide. (DOC 120 kb) [file 12909_2019_1679_MOESM2_ESM.doc]

**Faculty of Health Sciences**

**Socially Accountable Rural Placement Curriculum**

**Key Informant Questionnaire**

**Introduction:**

On behalf of the University Of Botswana Faculty Of Health Sciences, the investigator is conducting key informant surveys to learn about the community and field education of first and second year medical students at the University Of Botswana School Of Medicine, particularly in relation to their appreciation of needs of communities they will be interacting with. Your responses to the questionnaire will be used to identify potential areas for rural placement learning opportunities for the students. This questionnaire will take about 20-25 minutes of your time.

Your participation in this questionnaire is entirely voluntary. Your decision whether or not to participate will not affect you. Your responses will remain completely anonymous and no names or personal identifiers will be recorded or reported. We would like you to answer all of the questions as completely and honestly as you can. However, you may skip questions or decide not to complete the interview at any time.

**Type of respondent:** (Tick one and proceed to the page where that interview begins)

| **Respondent** | **Tick appropriate** |
| --- | --- |
| 1. □ Dean Faculty of Health Sciences | **2–3** |
| 1. □ Department head (SON, SOM, SPH) / Associate Dean, Associate Programmeme Director (FM, MMed, Community Nursing, Env. Health), I H S Deputy Principal, Faculty with CP | **4–6** |
| 1. □ Librarian | **8** |
| 1. □ IT or E learning staff | **9** |
| 1. □ Service provider - current or retired MOH Official | **10** |
| 1. □ Health professional Graduates within one (01) year of completion | **11** |
| 1. □ Curriculum Experts – UB, MOH | **12** |
| 1. □ Other (specify) |  |

**Part A: Dean Faculty o Health Sciences**

1. What are the minimum qualifications for each type of faculty member at your faculty? (Probes: Training and/or degree requirements, number of years of overall experience, type of experience (clinical/preceptor/classroom/lab teaching, etc.)

| **Faculty Title** | **Necessary Qualifications** |
| --- | --- |
| Lecturer |  |
| Senior Lecturer |  |
| Associate Professor |  |
| Head of Department |  |
| Professor |  |
| Other: |  |

1. Are there any faculty members who do not currently meet the minimum qualifications? Y/ N
   1. If yes, how many?
   2. Which qualifications are not being met?

__________________________________________________________________________________________________________________________________________________________________________________________________________________________________________________________________________________________________________________________________________________________________________________________________________________________

1. Do faculty qualification requirements differ by program? Y/ N
   1. If yes, please explain.

__________________________________________________________________________________________________________________________________________________________________________________________________________________________________________________________________________________________________________________________________________________________________________________________________________________________

1. Is there a standard faculty orientation process for new faculty members? Y/ N
   1. If yes, describe what it entails.

__________________________________________________________________________________________________________________________________________________________________________________________________________________________________________________________________________________________________________________________________________________________________________________________________________________________

1. Who determines what community placement-related content is included in each program’s curriculum?

__________________________________________________________________________________________________________________________________________________________________________________________________________________________________________________________________________________________________________________________________________________________________________________________________________________________

1. Do all departments have rural community placement program for their students? Y/ N.
   1. If yes, describe how each is implemented?

__________________________________________________________________________________________________________________________________________________________________________________________________________________________________________________________________________________________________________________________________________________________________________________________________________________________

1. What are the major teaching strengths in your institution?

__________________________________________________________________________________________________________________________________________________________________________________________________________________________________________________________________________________________________________________________________________________________________________________________________________________________

1. What are the most important needs to enhance and strengthen community placement training in preservice education?

__________________________________________________________________________________________________________________________________________________________________________________________________________________________________________________________________________________________________________________________________________________________________________________________________________________________

We’ve reached the end of the interview. Do you have any questions for me before we conclude?

Thank you for your time!

**Part B: Department Head (Department/ Programme Name: SOM, SON, SPH) / Ass. Prog Director**

1. What are the requirements for graduation from this programme? (Probes: skills assessment, knowledge assessment, etc.)

_________________________________________________________________________________________________________________________________________________________________________________________________________________________________________________________________________________________________________________________________________________________________________________________________________________________________________

1. What is the total number of students in your program (across all years)?
   1. How many students are there in year 1? (2011-2012 admission): ____________________
   2. Is the number of students in the programme staying the same, declining or increasing compared to the number in previous years? Please explain why you think this is.

_______________________________________________________________________________________________________________________________________________________________________________________________________________________________________________________________

1. What types of faculty members do you have in your programme (i.e. what are their titles)? (*Check titles that are mentioned).*
   1. How many faculty members of each title are there?
   2. How many faculty members of each title are currently teaching in other faculty programmes? (E.g., how many lecturers within your programme are teaching in other programmes)?

| **X** | **Faculty Title** | **Number of Faculty** | **Number of Faculty Teaching in Other Programmes** |
| --- | --- | --- | --- |
|  | Lecturer |  |  |
|  | Senior Lecturer |  |  |
|  | Associate Professor |  |  |
|  | Head of Department |  |  |
|  | Professor |  |  |
|  | Other: |  |  |
|  | **TOTAL NUMBER** |  |  |

1. Is there a ***programme-specific*** orientation process or mentoring programme for new faculty members? Y/ N
   1. If yes, describe what it entails.

____________________________________________________________________________________________________________________________________________________________________________________________________________________________________________________________________________________________________________________________________________________

1. What was the date of the most recent revision or update to your programme’s curriculum? ___________
2. How is community placement-related content currently taught in the programme’s curriculum? (Probes: in which years/ courses, what teaching methodologies are used, what are the needs of communities your students are placed in)?

_________________________________________________________________________________________________________________________________________________________________________________________________________________________________________________________________________________________________________________________________________________________________________________________________________________________________________

1. What does the “social accountability” component of the community placement programme within your programme entail? (Probe: what is the specific socially accountable field work? In what years/ classes is this practical component undertaken?)

_________________________________________________________________________________________________________________________________________________________________________________________________________________________________________________________________________________________________________________________________________________________________________________________________________________________________________

1. What would you say are the major strengths of the programme with regards to socially accountable -related content and teaching?

_________________________________________________________________________________________________________________________________________________________________________________________________________________________________________________________________________________________________________________________________________________________________________________________________________________________________________

1. Describe any gaps/missing areas in ***socially accountable rural community placement*** -related content or teaching within your programme.
   1. What can be done to address those gaps?

_________________________________________________________________________________________________________________________________________________________________________________________________________________________________________________________________________________________________________________________________________________________________________________________________________________________________________

1. Describe the ***socially accountable*** required materials used in this programme.
2. How do faculty access them? (Probe: receive free of charge, purchase them, borrow them, etc.)
3. How do students access them? (Probe: purchase them, copy them, use in or borrow from the library, use in the classroom, etc).

____________________________________________________________________________________________________________________________________________________________________________________________________________________________________________________________________________________________________________________________________________________

____________________________________________________________________________________________________________________________________________________________________________________________________________________________________________________________________________________________________________________________________________________

1. What types of ***socially accountable rural placement*** -related teaching and learning resources are available for faculty and students in the programme? (Probe: library print/ electronic/ online reference materials, audiovisual equipment, skills labs, Global / National Guidelines, national curricula used for ***socially accountable community placement*** related training of healthcare providers, etc).
   1. What proportion of faculty members would you say regularly use these resources? What proportion of students?
   2. Can you describe any issues with these resources? (Probe: currency, access, usage, functioning, etc).

_________________________________________________________________________________________________________________________________________________________________________________________________________________________________________________________________________________________________________________________________________________________________________________________________________________________________________

_______________________________________________________________________________________________________________________________________________________________________________________________________________________________________________________________

1. Describe the methods used for student ***socially accountable community placement*** knowledge and skill assessment throughout the programme.
2. Are both theory and practical/ clinical skills assessed? How?
3. Do students undergo a skills assessment before moving into the field practice setting? Please describe.
4. How students are assessed during their field/community placements?

____________________________________________________________________________________________________________________________________________________________________________________________________________________________________________________________________________________________________________________________________________________

___________________________________________________________________________________________________________________________________________________________________________________________________________________________________________________________________________________________________________________________________________________________________________________________________________________________________________________________________________________________________________________________________________________________________________________________________________________

1. Describe the rural placement sites used for community placement training of students.

(Probing questions):

1. How are they identified? (Probe: available preceptor, adequate space, similarity to post-graduation practice sites, mixed caseload, etc.)
2. How many sites do you place students at?
3. What is the role of students at these sites? (Probe: skill practice and development, observation, community diagnosis etc.)
4. Where are they located?
5. What types of sites are they (e.g. wards, villages, settlements, cattle posts, townships, town, and city)?

____________________________________________________________________________________________________________________________________________________________________________________________________________________________________________________________________________________________________________________________________________________

_______________________________________________________________________________________________________________________________________________________________________________________________________________________________________________________________

1. Describe how the programme uses field/ community preceptors.

(Probing questions):

- 1. Who is responsible for precepting and how are they identified? (Probe for any faculty role in precepting). Are there any required qualifications?
  2. Do they receive an orientation to their role and the programme prior to beginning?
  3. Do they receive any incentives or compensation for their role?
  4. What is their relationship with faculty?

____________________________________________________________________________________________________________________________________________________________________________________________________________________________________________________________________________________________________________________________________________________

____________________________________________________________________________________________________________________________________________________________________________________________________________________________________________________________________________________________________________________________________________________

We’ve reached the end of the interview. Do you have any questions for me before we conclude?

Thank you for your time!

**Part C: Librarian**

1. Describe the library resources on ***social accountability medical / health professions education*** available for faculty and students at your HTI. (Tick if mentioned)
2. □ Hard-copy reference materials (e.g., journals, textbooks, etc)
3. □ Electronic reference materials (e.g., CD-ROMs, DVDs, etc)
4. □ On-line journals
5. □ HIV and AIDS kiosks
6. □ Training/ tutorials (e.g., computer hardware, software, EBSCO-HOST, literature searching, etc)
7. □ Other (list):

____________________________________________________________________________________________________________________________________________________________________________________________________________________________________________________________________________________________________________________________________________________

1. Are faculty members and students regularly accessing these resources? Y/ N
   - 1. What proportion of faculty?
     2. What proportion of students?

____________________________________________________________________________________________________________________________________________________________________________________________________________________________________________________________________________________________________________________________________________________

1. What would you say are the major strengths of the ***social accountability medical / health professions education*** library resources at your university library?

_________________________________________________________________________________________________________________________________________________________________________________________________________________________________________________________________________________________________________________________________________________________________________________________________________________________________________

1. What would you say are the barriers to use of the ***social accountability medical / health professions education*** library resources? (Probe: currency (age of resources), access, usage, functioning, etc).

_________________________________________________________________________________________________________________________________________________________________________________________________________________________________________________________________________________________________________________________________________________________________________________________________________________________________________

We’ve reached the end of the interview. Do you have any questions for me before we conclude?

Thank you for your time!

**Part D: IT staff**

1. Describe the audio-visual resources available for teaching and learning ***social accountability medical / health professions education*** content at your HTI. (*Tick (√) the row next to the AV resources that are available*). (Probe: how many of each equipment type do you have? Where these resources are usually located and accessed?

| **√** | **AV resource** | **Number on campus** | **Usual location** |
| --- | --- | --- | --- |
|  | 1. LCD projector |  |  |
|  | 1. Laptop |  |  |
|  | 1. PA system |  |  |
|  | 1. DVD players |  |  |
|  | 1. TV |  |  |
|  | 1. VCR |  |  |
|  | 1. Other (list): |  |  |

_______________________________________________________________________________________________________________________________________________________________________________________________________________________________________________________________

1. Are faculty members and students regularly accessing these resources? Y/ N
   - 1. What proportion of faculty members?
     2. What proportion of students?
     3. What is the process for borrowing/ using these resources?

_______________________________________________________________________________________________________________________________________________________________________________________________________________________________________________________________

1. Describe any barriers to use of the AV resources? (Probe: age of equipment, access, usage, functioning, etc).

_______________________________________________________________________________________________________________________________________________________________________________________________________________________________________________________________4. What would you recommend for students and faculty interaction while on community placement?

_______________________________________________________________________________________________________________________________________________________________________________________________________________________________________________________________

We’ve reached the end of the interview. Do you have any questions for me before we conclude?

Thank you for your time!

**Part E: Service providers (Supervisor/ Nurse Administrator, Current or retired MOH officers etc)**

1. Describe how health professionals’ practical training is provided/ conducted at your health district.

________________________________________________________________________________________________________________________________________________________________________________________________________________________________________________________________________________________________________________________________________________________________________________________________________________________________________________________________________________________________________________________________________________________________________________________________________________________________________________________________________________________________________

1. What would you say are the major strengths of new health professions graduates (Nurse, Env. Officer, Health Educator, Dr...etc) who begin work at your facility, are in their ability to meet the needs of communities they interact with?

________________________________________________________________________________________________________________________________________________________________________________________________________________________________________________________________________________________________________________________________________________________________________________________________________________________________________________________________________________________________________________________________________________________________________________________________________________________________________________________________________________________________________

1. What gaps have you identified in the knowledge and skills of health professions graduates now working at your facility regarding identifying the needs of communities they serve as well as addressing them? Please describe. (Probe for knowledge or skill deficits (competencies, provider-client interaction, etc).

_______________________________________________________________________________________________________________________________________________________________________________________________________________________________________________________________4. What should be done to address those gaps?

_______________________________________________________________________________________________________________________________________________________________________________________________________________________________________________________________

We’ve reached the end of the interview. Do you have any questions for me before we conclude?

Thank you for your time!

**Part F: Graduates within one year post completion (Medical interns, nurses, Env Health Officers...)**

1. What do you consider to be the strengths of the community-related training you received at your preservice institution? (Alternate phrasing: In what way do you think your training at your preservice institution prepared you for your community-related work? Probes: Hygienic practices, waste management, PMTCT, HIV counselling and testing, lifestyle, poverty eradication etc).

______________________________________________________________________________________________________________________________________________________________________________________________________________________________________________________________________________________________________________________________________________________________________________________________________________________________________________________________________________________________________________________________

1. What do you consider to be gaps or missing areas in the community-related training you received at your preservice institution? (Alternate phrasing: After entering the workforce, in what areas of community-related work did you not feel fully prepared?)

______________________________________________________________________________________________________________________________________________________________________________________________________________________________________________________________________________________________________________________________________________________________________________________________________________________________________________________________________________________________________________________________

1. What are your challenges in addressing clients’ community needs?

____________________________________________________________________________________________________________________________________________________________________________________________________________________________________________________________________________________________________________________________________________________

1. How can community-related teaching and learning be improved?
   1. What might have helped you to better learn the community-related knowledge and skills you need for your job?

_________________________________________________________________________________________________________________________________________________________________________________________________________________________________________________________________________________________________________________________________________________________________________________________________________________________________________

We’ve reached the end of the interview. Do you have any questions for me before we conclude?

Thank you for your time!

**Part G: Curriculum Experts_UB, MOH**

1. What do you consider to be the strengths of the community placement-related training students receive from the faculty of health sciences **or** UB_SOM?

______________________________________________________________________________________________________________________________________________________________________________________________________________________________________________________________________________________________________________________________________________________________________________________________________________________________________________________________________________________________________________________________

**2.** What do you consider to be gaps or missing areas in the community placement -related training offered at the faculty of health sciences?

___________________________________________________________________________________________________________________________________________________________________________________________________________________________________________________________________________________________________________________________________________________________________________________________________________________________________________________________________________________________________________________________________________________________________________________________________________________

3. How can the Faculty of Health Sciences’ socially accountable community placement -related teaching is improved?

______________________________________________________________________________________________________________________________________________________________________________________________________________________________________________________________________________________________________________________________________________________________________________________________________________________________________________________________________________________________________________________________

1. What are the challenges in incorporating new socially accountable community placement content into the various Faculty of Health Sciences curricula?
   1. What is the process for identifying and incorporating new academic content?
   2. How often are the curricula revised and updated?

______________________________________________________________________________________________________________________________________________________________________________________________________________________________________________________________________________________________________________________________________________________________________________________________________________________________________________________________________________________________________________________________

1. What should be considered as key areas to consider in designing an appropriate socially accountable programme for 1^st^ and 2^nd^ year medical students.

______________________________________________________________________________________________________________________________________________________________________________________________________________________________________________________________________________________________________________________________________________________________________________________________________________________________________________________________________________________________________________________________

We’ve reached the end of the interview. Do you have any questions or comments for me before we conclude?

Thank you for your time!
